# Understanding physical activity interventions for people living with dementia: A systematic review and meta‐analysis

**DOI:** 10.1002/alz70858_105729

**Published:** 2025-12-25

**Authors:** Laura E. Middleton, Liu Grace, Vanessa Vucea‐Tirabassi, Christine Aiken, Jennifer Bethell, Heather A. Cooke, Heather Keller, Teresa Liu‐Ambrose, Myrna Norman, Megan E O'Connell, Jackie E Stapleton, Ingrid Waldron, Sarah A. Wu, Marie‐Lee Yous, Carrie A McAiney

**Affiliations:** ^1^ Schlegel‐UW Research Institute for Aging, Waterloo, ON, Canada; ^2^ University of Waterloo, Waterloo, ON, Canada; ^3^ University of Toronto, Toronto, ON, Canada; ^4^ KITE Research Institute, Toronto Rehabilitation Institute ‐ University Health Network, Toronto, ON, Canada; ^5^ Alzheimer Society of B.C., Vancouver, BC, Canada; ^6^ Vancouver Coastal Health Research Institute, Vancouver, BC, Canada; ^7^ Centre for Aging SMART, Vancouver Coastal Health Research Institute, Vancouver, BC, Canada; ^8^ University of British Columbia, Vancouver, BC, Canada; ^9^ Djavad Mowafaghian Centre for Brain Health, Vancouver, BC, Canada; ^10^ University of Saskatchewan, Saskatoon, SK, Canada; ^11^ McMaster University, Hamilton, ON, Canada

## Abstract

**Background:**

There is limited research focused on lifestyle interventions among people living with dementia with recent systematic reviews primarily focused on the impact of exercise on cognition. However, functional abilities and quality of life (QoL) are the outcomes most consistently prioritized by people living with dementia, care partners, and healthcare professionals. We conducted a systematic review to understand the impact of two lifestyle interventions (physical activity, nutrition) on the functional abilities, QoL, and nutritional status (nutrition interventions only) of people with dementia living in the community. The findings here focus on the effects of physical activity interventions.

**Method:**

Systematic literature searches for peer‐reviewed intervention studies were conducted in four databases (MEDLINE, EMBASE, Scopus, CINAHL). Articles were exported to Covidence, where duplicates were removed and two independent reviewers performed study selection, data extraction, and risk of bias assessments. A narrative synthesis was conducted. A meta‐analysis is currently ongoing.

**Result:**

Fifty‐five studies met the inclusion criteria and were included in this review. Data extraction revealed diverse study designs, characteristics and types of physical activity interventions, and measures for functional abilities and QoL. Nine studies measured the QoL of people living with dementia using the self‐reported Quality of Life Alzheimer's Disease (QoLAD) tool. Four of these studies showed a significant improvement in the QoLAD. Twenty‐five studies reported using the Timed Up and Go Test (TUG) to measure mobility. Fifteen of these studies demonstrated an improvement in the TUG, with nine of the studies revealing significant improvements. Six studies measured functional capacity using the 6‐minute walk test. Two of these studies showed a significant improvement in the 6‐minute walk test.

**Conclusion:**

The results from this review highlight the impact of physical activity interventions on QoL and mobility for people living with dementia. The meta‐analysis will provide quantitative estimates for the effects of physical activity interventions on specific outcomes related to functional abilities and QoL. Furthermore, exploring the effectiveness by intervention dose and delivery in subgroup analyses will also allow us to better inform guidelines regarding interventions, programs, and policy related to physical activity interventions. Lastly, we will identify knowledge gaps where research is needed.

